# Arrhythmogenic substrate at the interventricular septum as a target site for radiofrequency catheter ablation of recurrent ventricular tachycardia in left dominant arrhythmogenic cardiomyopathy

**DOI:** 10.1186/s12872-015-0010-8

**Published:** 2015-03-10

**Authors:** Stepan Havranek, Tomas Palecek, Tomas Kovarnik, Ivana Vitkova, Miroslav Psenicka, Ales Linhart, Dan Wichterle

**Affiliations:** 2nd Department of Medicine – Department of Cardiovascular Medicine, First Faculty of Medicine, Charles University and General University Hospital in Prague, U Nemocnice 2, Prague, 128 08 Czech Republic; Institute of Pathology, First Faculty of Medicine, Charles University and General University Hospital in Prague, Studnickova 2, 128 00 Prague, Czech Republic; Department of Cardiology, Institute for Clinical and Experimental Medicine, Videnska 1958/9, Prague, 140 21 Czech Republic

**Keywords:** Left dominant arrhythmogenic cardiomyopathy, Ventricular tachycardia, Magnetic resonance imaging, Endomyocardial biopsy, Catheter ablation

## Abstract

**Background:**

Left dominant arrhythmogenic cardiomyopathy (LDAC) is a rare condition characterised by progressive fibrofatty replacement of the myocardium of the left ventricle (LV) in combination with ventricular arrhythmias of LV origin.

**Case presentation:**

A thirty-five-year-old male was referred for evaluation of recurrent sustained monomorphic ventricular tachycardia (VT) of 200 bpm and right bundle branch block (RBBB) morphology. Cardiac magnetic resonance imaging showed late gadolinium enhancement distributed circumferentially in the epicardial layer of the LV free wall myocardium including the rightward portion of the interventricular septum (IVS). The clinical RBBB VT was reproduced during the EP study. Ablation at an LV septum site with absence of abnormal electrograms and a suboptimum pacemap rendered the VT of clinical morphology noninducible. Three other VTs, all of left bundle branch block (LBBB) pattern, were induced by programmed electrical stimulation. The regions corresponding to abnormal electrograms were identified and ablated at the mid-to-apical RV septum and the anteroseptal portion of the right ventricular outflow tract. No abnormalities were found at the RV free wall including the inferolateral peritricuspid annulus region. Histological examination confirmed the presence of abnormal fibrous and adipose tissue with myocyte reduction in endomyocardial samples taken from both the left and right aspects of the IVS.

**Conclusion:**

LDAC rarely manifests with sustained monomorphic ventricular tachycardia. In this case, several VTs of both RBBB and LBBB morphology were amenable to endocardial radiofrequency catheter ablation.

## Background

Left dominant arrhythmogenic cardiomyopathy (LDAC) has been recently introduced as a rare condition characterised by progressive fibrofatty replacement exclusive to the myocardium of the left ventricle (LV) in combination with ventricular arrhythmias of LV origin [1-9]. Sustained ventricular tachycardia (VT) has been observed rarely and catheter ablation for VT in an LDAC patient has never been reported.

## Case presentation

**Figure 1:**
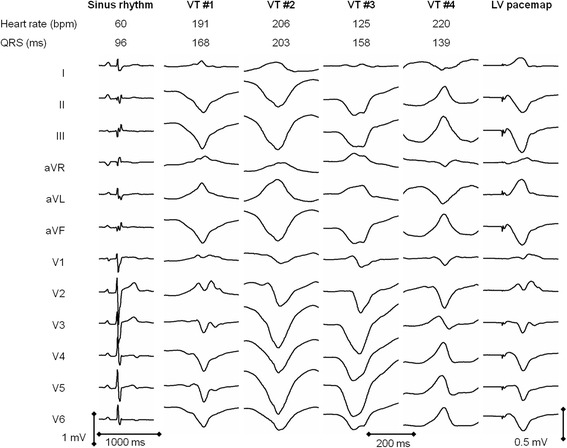
**ECG in sinus rhythm, clinical ventricular tachycardia (VT #1) and three other induced VT morphologies during the electrophysiological procedure (VT #2 – #4).** The pacemap for VT #1 is shown in the last column corresponding to the site marked by an asterisk in Figure 3.

**Figure 2:**
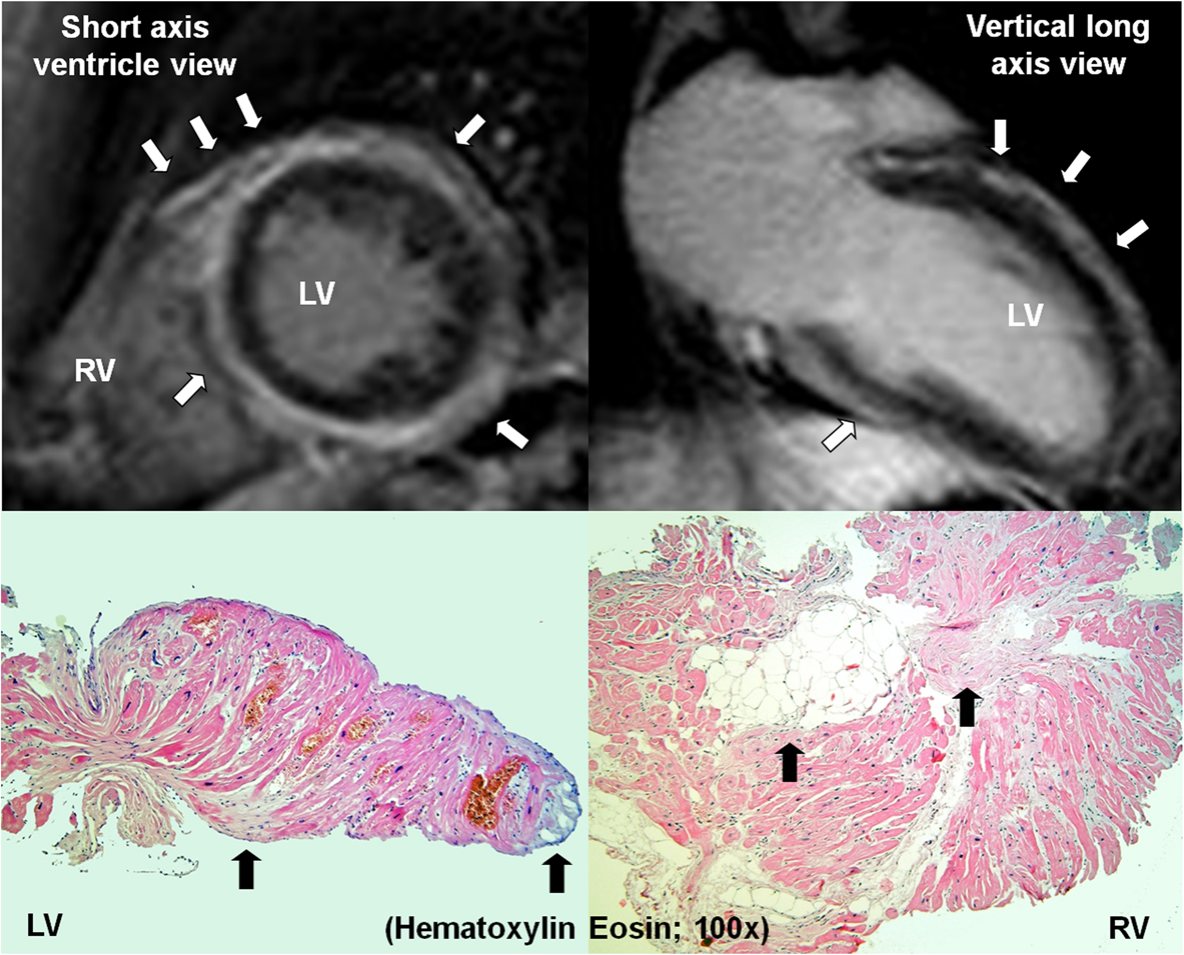
**Distribution of late gadolinium enhancement (arrows) in short axis (left upper panel) and vertical long axis view (right upper panel) was distributed circumferentially in the subepicardial left ventricular free wall myocardium with discrete progression to the adjacent mid-anterior free wall of the right ventricle.** Note the subendocardial involvement at the RV aspect of the interventricular septum. Histological assessment of endomyocardial samples: from the left ventricular site of abnormal electrograms indicated by the cross in Figure 3 (left bottom panel); from right ventricular aspect of the interventricular septum (right bottom panel). Arrows indicate abnormal fibrosis and adipose tissue. Staining: hematoxylin-eosin. Magnified 100 times.

A thirty-five-year-old male was referred for evaluation of recurrent hemodynamically tolerated sustained monomorphic ventricular tachycardia of 200 bpm, which had right bundle branch block (RBBB) morphology with leftward axis deviation (Figure 1). He suffered from non-syncopal palpitations in the past 3 months. He was in functional class NYHA I and had no symptoms suggestive of ischemic heart disease. His medical history was unremarkable. There was no family history of cardiomyopathies or sudden unexplained death. His 12-lead ECG in sinus rhythm was clearly abnormal with borderline Q-waves in the inferior leads, mid-QRS notching and slurring of narrow QRS complexes (QRSd of 98 ms) in limb and right precordial leads, respectively, and flattened biphasic or negative T waves in the inferolateral leads (Figure 1). Echocardiography detected slight LV dilatation (end-diastolic diameter of 63 mm) with mild global hypokinesia (ejection fraction of 42%). CT coronary angiography excluded coronary artery disease. Cardiac magnetic resonance imaging (CMR) showed late gadolinium enhancement (LGE), which was distributed circumferentially in the epicardial layer of the LV free wall myocardium (approximately one-third of the LV wall thickness) including the rightward portion of the interventricular septum (IVS) (Figure 2). The LGE spread also to a small adjacent region of the mid-anterior free wall of right ventricle (RV). Moreover, T1-weighted and SPIR magnetic resonance sequences visualised adipose infiltration of myocardium in the anterior right IVS and an adjacent portion of the anterior RV free wall in the zone of positive LGE. The LV was slightly dilated (end-diastolic diameter of 62 mm, end-diastolic volume of 287 mL) with mild global hypokinesia (ejection fraction of 50%). There were no wall motion abnormalities of the RV. The typical scar distribution together with ECG abnormalities and VT of RBBB morphology suggested the diagnosis of LDAC. Treatment with bisoprolol 2.5 mg and trandolapril 4 mg daily was initiated.

**Figure 3:**
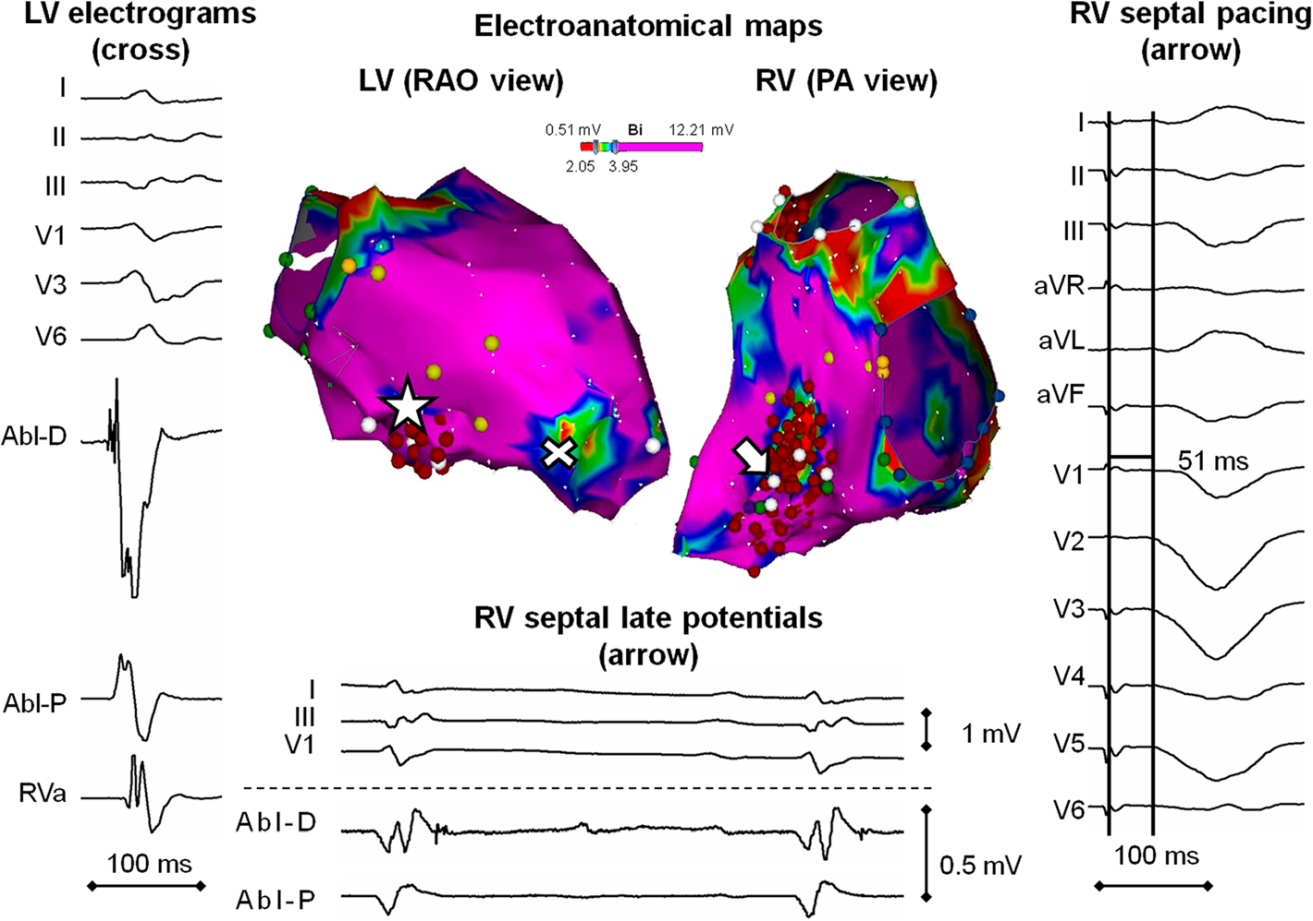
**The bipolar voltage map of both ventricles in sinus rhythm.** An atypical range (2–4 mV) for color-coding was used to highlight the areas of subtle reduction of bipolar voltages. The cross denotes the left ventricular apicoseptal region of abnormal electrograms where the endomyocardial biopsy was taken. The arrow indicates the site with a maximum stimulus-to-QRS interval at the right-sided interventricular septum. The asterisk shows the site of the pacemap for the clinical tachycardia at the left-sided interventricular septum site with normal electrograms.

Because of recurrent VT despite medical therapy, an electrophysiological study, 3D electroanatomical mapping, and ablation were indicated. The procedure was performed under local anesthesia and mild conscious sedation with midazolam and alfentanyl. At the beginning of the procedure, clinical VT of 191 bpm and RBBB morphology (Figure 1, VT #1) was induced by programmed LV stimulation. This was unexpectedly poorly tolerated, so the procedure was continued with substrate mapping in sinus rhythm (3.5 mm irrigated-tip catheter, NaviStar Thermocool, D-curve and CARTO 3, Biosense Webster Inc., Diamond Bar, CA, USA). The procedure was guided by intracardiac echocardiography (AcuNav Diagnostic Ultrasound Catheter, Acuson – Siemens, Mountain View, CA, USA). The LV was accessed by a transseptal approach using a steerable sheath (Agilis, St. Jude Medical Inc., St. Paul, MN, USA) in order to enable subsequent endomyocardial biopsy. As expected, endocardial LV bipolar mapping did not reveal low-voltage areas <1.5 mV. There were a limited number of sites with abnormal electrograms, which were located only at the apicoseptal LV region (Figure 3). Stimulus-to-QRS (S-QRS) delay did not exceed 20 ms in any of them. The best, but far from optimum, pacemap (Figure 1) for the clinical VT was achieved in the mid-inferior LV septum where the morphology of bipolar electrograms was fairly normal. Despite this finding, RF energy (Stockert EP Shuttle, Biosense Webster Inc., Diamond Bar, CA, USA; setting: 30 W, <40°C, 30 ml/min) was delivered at this site and its close vicinity. After the initial ablation, we were unable to re-induce the VT of clinical morphology. However, three other non-clinical VTs (of 206, 125 and 220 bpm), all of left bundle branch block (LBBB) pattern, were observed (Figure 1, VT #2 - #4). They were inducible by programmed stimulation from the RV/LV or by catheter manipulation and were either non-sustained or non-tolerated. The pacemapping for VT morphology #2 and #3 suggested an exit site at the mid-to-apical RV septum. VT morphology #4 had an inferior axis and its exit site was located at the anteroseptal portion of the right ventricular outflow tract (RVOT). Electroanatomical mapping of the RV identified dispersed regions of abnormal electrograms, predominantly at the septum and the anterior/septum portions of the RVOT, while the RV free wall including the inferolateral peritricuspid annulus region was generally not affected. Maximum scar involvement was found at the RV aspect of the IVS, where sites with discrete late potentials and slow conduction zones (maximum S-QRS of 50 ms) were identified (Figure 3). Substrate-based ablation was performed rather extensively at the RV aspect of the midseptum from the inferior to middle segments up to the proximity of right bundle branch (contralateral to the exit of clinical VT #1 and close to the suspected exits of VT #2 and #3) as well as at the anterior portion of the RVOT (site of origin of VT #4).

An endomyocardial biopsy was performed prior to ablation, with an attempt to guide the catheter bioptome (7-F/104 cm biopsy forceps, Cordis, Bridgewater, NJ, USA) into affected regions according to electroanatomical mapping. Histological examination confirmed the presence of abnormal fibrous and adipose tissue with myocyte reduction in endomyocardial samples taken from both left and right aspects of the IVS (Figure 2). Mutation screening for desmosomal genes was not performed.

A cardioverter-defibrillator (ICD) was implanted and the patient was discharged on the same dosage of bisoprolol and trandolapril medication. No class I or III antiarrhythmic drugs were given. During the 18-month follow-up, the patient experienced only a single episode of monomorphic VT (243 bpm) when bisoprolol medication was discontinued by mistake. LV dilatation and mild dysfunction remained stable at regular check-ups.

## Discussion

The phenotypic spectrum of arrhythmogenic cardiomyopathy includes either left dominant, right dominant or bi-ventricular variant. Isolated left and right ventricular abnormalities are at two extremes of the clinical manifestation of the disease [8]. There were no specific ECG abnormalities in the right precordial leads in our patient. The RV was not dilated and had normal kinetics. Typical regions involved in classic arrhythmogenic right ventricular cardiomyopathy (ARVC) (e.g. inferolateral peri-tricuspid area) were free of fibrofatty replacement according to both LGE CMR and electroanatomical mapping. The diagnosis of LDAC in this case was based on recently established clinical features of the disease [8]: ventricular arrhythmia of LV origin, repolarization abnormalities in inferolateral leads, mild LV dilatation with systolic dysfunction, and myocardial fibrofatty replacement assessed by LGE on CMR and confirmed by endomyocardial biopsy.

The designation as a purely LV entity might be questioned because a small region of fibrofatty replacement was present at the mid-anterior portion of the RV free wall, as assessed by LGE CMR. An additional region of abnormal electrograms was present at the anterior RVOT by electroanatomical mapping (not revealed by CMR), complemented by the observation that inducible VT #4 exited from this site. This is still compatible with LDAC because LV involvement including IVS clearly dominated and subtle RV regional abnormalities or the presence of additional arrhythmogenic substrate in RV has been observed in the majority of LDAC patients [8].

Although the presence of ventricular arrhythmias is an essential component of the clinical picture of LDAC, sustained monomorphic VT has been reported rarely. Besides the cases diagnosed post-mortem, the predominant arrhythmias were frequent ventricular premature beats or non-sustained VT of LV origin [8]. Sustained VT of RBBB morphology has been described only in a minority of patients: in 1 of 42 patients in Sen-Chowdry’s cohort [8], in 3 of 7 carriers of a dominant desmoplakin mutation in single family [5] and in 3 more solitary case reports [3,10,11]. To the best of our knowledge, catheter ablation has never been performed for VT in an LDAC patient.

Similarly to classic ARVC, catheter ablation therapy in LDAC may be indicated in patients with recurrent VT despite therapy with antiarrhythmic drugs. In this case we preferred non-pharmacological treatment because of the considerable burden of arrhythmia, in order to prevent frequent ICD therapy and to avoid long-term medical therapy, which may be ineffective and/or associated with adverse effects.

Because of the non-transmural and rather homogeneous distribution of pathological tissue, electroanatomical mapping (both bipolar and unipolar) showed voltages almost invariably within the normal arbitrary range and thus was of little help. For this reason pre-procedural CMR was extremely valuable for tailoring the ablation strategy. It demonstrated from the very beginning that the clinical VT could only be effectively targeted by endocardial ablation from the RV aspect of IVS - the only site where the potential substrate was localized close to the endocardial surface. Any other LV targets would require an epicardial approach. Fortunately, VT #1 - #3 originated at the IVS. It is likely that the clinical VT (VT #1) originated from the RV subendocardium at the IVS with a preferential leftward exit, which could explain the RBBB morphology. Initial ablation at the LV aspect of the IVS probably modified the route of propagation. This explains why subsequent VT #2 and #3 had LBBB morphology. It is not known whether the initial LV ablation had any other impact besides changing the exit route.

Indeed, there was a preponderance of mappable arrhythmogenic substrate at the RV aspect of the IVS, although in sinus rhythm the abnormal electrograms were considerably masked by the fast activation of the septum from both sides via the intact His-Purkinje system conduction. Pacing from the LV would probably be helpful maneuver to reveal pathological electrograms or late potentials at the right side of the IVS, but this was not done in the present case. Ablation at the RV aspect of the IVS was performed rather extensively to target not only the critical zones of inducible VTs but also to homogenize the scar in order to prevent other VTs that did not manifest during the procedure.

Although the patient presented with VT of RBBB morphology, this case also suggests that, at least in theory, LDAC may manifest by ventricular arrhythmias of LBBB morphology alone. This might be taken into account when the clinical criteria of LDAC are refined in the future.

Endomyocardial biopsy guided by electroanatomical mapping confirmed the presence of fibrous and adipose tissue in samples obtained from both aspects of the IVS. Positive sampling from the right-sided IVS, fully affected by the pathological process, was expected in our case. The biopsy from the LV endomyocardium, without macroscopic signs of pathology by LGE CMR, was taken from the apicoseptal region of LV, which was the only site with borderline abnormal electrograms. Such an approach could possibly increase the diagnostic yield, as already reported [12].

Because of structural heart disease that manifested by malignant ventricular arrhythmia and the existence of large-scale, potentially arrhythmogenic substrate, which could not be safely eliminated by catheter ablation, and because of the progressive nature of disease, the cardioverter-defibrillator was implanted for secondary prevention of sudden cardiac death. Despite the fact that single episode of VT was documented during the follow-up, it is likely that catheter ablation prevented frequent VT recurrences.

## Conclusion

Left dominant arrhythmogenic cardiomyopathy is an infrequent structural heart disease, which rarely manifests with sustained monomorphic ventricular tachycardia. In this case, extensive circumferential LV involvement by fibrofatty replacement was distributed epicardially at the LV free wall and subendocardially at the RV aspect of the interventricular septum. Several VTs of both RBBB and LBBB morphology were amenable to endocardial radiofrequency catheter ablation.

## Consent

Written informed consent was obtained from the patient to the publication of this case report and any accompanying images. A copy of the written consent is available for review by the Editor of this journal.

## Abbreviations

LDAC: Left dominant arrhythmogenic cardiomyopathy
LV: Left ventricle
VT: Ventricular tachycardia
RBBB: Right bundle branch block
CMR: Cardiac magnetic resonance
LGE: Late gadolinium enhancement
IVS: Interventricular septum
RV: Right ventricle
S-QRS: Stimulus-to-QRS
LBBB: Left bundle branch block
RVOT: Right ventricular outflow tract
ARVC: Arrhythmogenic right ventricular cardiomyopathy
ICD: Implantable cardioverter-defibrillator
